# The Impact of Cognitive Flexibility and Cognitive Control on Turnover Intention: The Roles of Impulsivity and Anger Expression Among Emergency Nurses

**DOI:** 10.1155/jonm/6503308

**Published:** 2026-08-04

**Authors:** Nukhet Bayer, Ayşegül Turan

**Affiliations:** ^1^ Faculty of Health Sciences, Nursing Department, Lokman Hekim University, Ankara, Türkiye, lokmanhekim.edu.tr; ^2^ Faculty of Health Sciences, Nursing Department, Kırşehir Ahi Evran University, Kırşehir, Türkiye, lokmanhekim.edu.tr

**Keywords:** anger, cognitive control, cognitive flexibility, emergency department nurse, impulsivity, intention to leave

## Abstract

**Objective:**

The objective of this study is to examine the relationships between cognitive flexibility, cognitive control, impulsivity and anger expression and intention to leave among emergency department nurses.

**Method:**

This descriptive and cross‐sectional study was conducted with 295 nurses working in the emergency departments of seven hospitals in Ankara for at least 1 year. Data were collected using a personal information form, the Impulsivity Scale, the Cognitive Control and Cognitive Flexibility Scale, the Anger Expression Scale and the Job Intention Scale. Descriptive statistics, independent group *t*‐test, one‐way analysis of variance (ANOVA), Pearson correlation and multiple regression analyses were used.

**Results:**

Regression analysis revealed that impulsivity, anger expression style and cognitive control–flexibility together accounted for 5.3% of the variance in intention to leave, suggesting that additional organisational and environmental factors likely contribute to turnover intention. Impulsivity (*β* = 0.138) and anger expression level (*β* = 0.131) were positively associated with intention to leave, whereas higher cognitive control and flexibility were negatively associated with intention to leave (*β* = −0.121). Correlation analyses similarly indicated significant positive associations between impulsivity and anger expression and intention to leave and significant negative associations between cognitive control and flexibility and intention to leave.

**Conclusion:**

The findings suggest that impulsivity and anger expression patterns among emergency department nurses are associated with higher intention to leave. Cognitive control and cognitive flexibility may serve as important psychological resources potentially related to reduced turnover intention. Interventions aimed at strengthening nurses’ cognitive and emotional regulation skills in high‐stress work environments such as emergency departments may contribute to workforce continuity; however, given the cross‐sectional design, causal interpretations should be made with caution.

**Implications for Nursing Management:**

Emergency department nurse managers should consider strategies that enhance nurses’ cognitive control and cognitive flexibility as potential resources against turnover intention. Structured interventions such as stress management programs, emotional regulation training and leadership practices that foster supportive and psychologically safe work environments may help mitigate impulsivity and maladaptive anger expression. Integrating these approaches into workforce management and professional development plans could contribute to improved staff retention and organisational stability in high‐pressure clinical settings.

## 1. Introduction

Emergency departments are clinical settings characterised by high levels of work demand due to unpredictable patient numbers, traumatic cases and exposure to violence. Previous studies have shown that nurses working in high‐risk units such as emergency departments are frequently exposed to verbal and physical violence, and that workplace violence has a significant impact on emotional labour and intention to leave the job [1–3]. Furthermore, high workload and traumatic exposure have been reported to increase burnout, and burnout has been shown to increase intention to leave [4]. These conditions create a significant cognitive and emotional burden on nurses [5, 6]. Nurse retention is influenced by a complex interplay of organisational, psychological and work‐environment factors, and evidence suggests that these determinants are particularly salient in high‐pressure clinical settings such as emergency departments [7]. Therefore, it is important for nurses to be able to use high levels of cognitive control and cognitive flexibility to protect their mental and physical health [8].

Cognitive flexibility and cognitive control refer to the mental processes that enable individuals to focus their attention, suppress impulsive responses and adapt to rapidly changing situations [9, 10]. Within the framework of the Job Demands‐Resources (JD‐R) Model, these capacities function as personal psychological resources that buffer the adverse effects of high job demands, sustain adaptive coping and support the regulation of impulsive emotional responses [11]. When cognitive control is weak, individuals may exhibit increased impulsive behaviour, which can make it difficult to regulate emotional responses [12]. These skills are critical for effective decision‐making and adaptation, particularly in complex and uncertain environments [13]. In high‐stress work environments where workplace violence occurs, insufficient cognitive capacity can lead to increased levels of anger and its expression in dysfunctional ways [14, 15].

The uncontrolled expression or suppression of anger can negatively affect interpersonal relationships and lead to reduced job satisfaction and resignations [16–18]. Developing strategies to retain emergency nurses is a critical necessity for the sustainability of the healthcare system. The increased burnout and intention to leave the profession, particularly after Covid‐19, has exacerbated the shortage of nurses globally [19]. In Turkey, the number of nurses is also quite low compared to OECD countries, with approximately 3 nurses per 1000 people [20]. Therefore, ensuring the retention of nurses in units with high workloads and intense stress levels, such as emergency departments, is of great importance.

Various studies in the literature have identified that workplace violence among emergency department nurses increases anger, and that anger is associated with burnout and intention to leave [3, 21, 22]. However, the existing literature has predominantly examined these variables in isolation, and empirical evidence simultaneously investigating the combined role of cognitive flexibility, cognitive control, impulsivity and anger expression as predictors of turnover intention among emergency department nurses remains limited. The simultaneous examination of these interrelated psychological variables may offer a more comprehensive and theoretically grounded basis for developing targeted workforce retention strategies in emergency settings. According to the JD‐R Model, high job demands deplete employees’ energy resources and increase the risk of burnout and intention to leave [11], while personal psychological resources such as cognitive flexibility and cognitive control may facilitate adaptive coping and the regulation of anger‐related responses. Therefore, cognitive flexibility and cognitive control are considered important psychological resources that can buffer the impact of impulsivity and maladaptive anger expression on turnover intention in units with high job demands, such as emergency services.

### 1.1. Aim of the Study

This study aims to examine the relationships between cognitive flexibility, cognitive control, impulsivity, anger expression and intention to leave among nurses working in emergency departments. Within the framework of the JD‐R Model, cognitive flexibility and cognitive control are conceptualised as personal psychological resources that may moderate the adverse effects of impulsivity and maladaptive anger expression on turnover intention. The findings are intended to contribute to the development of evidence‐based, psychologically informed strategies for nursing workforce retention in high‐pressure clinical settings.

## 2. Methods

### 2.1. The Population and Sample of the Study

This study was designed and reported in accordance with the STROBE (Strengthening the Reporting of Observational Studies in Epidemiology) guidelines [23]. This descriptive and cross‐sectional study was conducted in seven different healthcare institutions located in Ankara, the capital city of Turkey, including the Education and Research Hospital, a state hospital and a university hospital. A power analysis was performed to determine the sample size to be included in the study. The power of the test was calculated using the G∗Power 3.1.9 programme. In a similar study in the literature by Zhu and Zhang [24], the effect size between anger and intention to leave the job was reported as 0.092. Accordingly, it was calculated that at least 144 participants were required to achieve a power level above 95% at a 5% significance level and an effect size of 0.092 (df = 142; *F* = 3.908). Although the power analysis was based on a previously reported effect size for the anger–turnover intention relationship [24], the final regression model incorporated multiple predictors. The achieved sample size of 295 was considered sufficient to detect small‐to‐medium effects in multiple regression analyses. Data for the study were collected between August and December 2022. A purposive sampling method was used in the study. The researchers distributed a total of 350 data collection forms to nurses. The inclusion criteria were having a minimum of 1 year of experience in the emergency department and providing voluntary consent to participate. Participants were given sufficient time to complete the forms, and the forms were collected by the researchers at the end of the specified period. The collected data forms were reviewed, and 55 forms that were left blank, incomplete, or incorrectly filled out were excluded from the study. The final sample consisted of 295 nurses. Although data were collected from a single city, the study was conducted across seven different healthcare institutions of varying types in Ankara, the capital city of Turkey.

### 2.2. Data Collection Tool

Research data were collected using a form targeting nurses’ individual and professional characteristics, along with four validated scales: the Impulsivity Scale, the Cognitive Control and Cognitive Flexibility Scale, the State‐Trait Anger Expression Inventory (STAXI) and the Intention to Leave Scale (ITLS).

#### 2.2.1. Cognitive Control and Cognitive Flexibility Scale

This scale was developed by Gabrys and colleagues [25]. The Turkish adaptation was carried out by Demirtaş [26]. The scale consists of 18 items and 2 subscales. These subscales are referred to as assessment and coping flexibility (9 items). The scale is a 7‐point Likert scale. The Cronbach’s alpha reliability coefficients for the scale are reported as 0.89 and 0.93 for the assessment and coping flexibility subdimensions, and 0.90 for the cognitive control over emotions subdimension. In the current study, the Cronbach’s alpha coefficient for this scale was found to be *α* = 0.908.

#### 2.2.2. ITLS

This scale was developed by Mobley et al. [27]. The Turkish adaptation and validity and reliability of the scale were determined by Örücü and Özafşarlioğlu [28]. The scale consists of 3 items and is a 5‐point Likert scale. The Cronbach’s alpha reliability coefficient of the scale was found to be 0.904. In the current study, the Cronbach’s alpha coefficient for this scale was found to be *α* = 0.884.

#### 2.2.3. STAXI

This scale was developed by Spielberger et al. [29]. The Turkish adaptation was carried out by Özer [30]. The scale consists of 34 items and is a 4‐point Likert scale. The scale consists of three subscales. The Cronbach’s alpha values, reported by Özer [30], ranged from 0.80 to 0.90 for the anger control subscale, 0.69 to 0.91 for the anger‐externalisation subscale and 0.58 to 0.76 for the anger internalisation subscale.

#### 2.2.4. Barratt Impulsiveness Scale (BIS‐11‐SF)

BIS‐11 was originally developed by Patton et al. [31]. The short form of the scale (BIS‐11‐SF) was later developed by Spinella [32] and adapted into Turkish by Güleç et al. [33]. The scale consists of 15 items and rated on a 4‐point Likert scale. It comprises three subdimensions: nonplanning impulsivity, motor impulsivity and attentional impulsivity. In the Turkish validation study conducted by Güleç et al. [33], the overall Cronbach’s alpha coefficient was reported as *α* = 0.82. In the current study, the Cronbach’s alpha coefficient for this scale was found to be *α* = 0.713.

### 2.3. Data Analysis

The data obtained in the study were evaluated using the SPSS 22.0 statistical programme. Frequency and percentage analyses were used to determine the descriptive characteristics of the nurses participating in the study, while mean and standard deviation statistics were used to examine the scale. Normality was assessed by examining skewness and kurtosis values, and results indicated that the variables met the assumptions of normal distribution. Parametric methods were used in the analysis of the data. The relationships between the dimensions determining the nurses’ scale levels were examined using Pearson correlation and multiple regression analyses. Independent group *t*‐tests and one‐way analysis of variance (ANOVA) were used to examine the differences in scale levels according to the nurses’ descriptive characteristics. In regression analysis, variance inflation factor (VIF) and tolerance values were used to assess model assumptions for multicollinearity, and the Durbin–Watson test was used to examine autocorrelation. All procedures were conducted in accordance with ethical standards approved by the Lokman Hekim University Ethics Committee (Decision No: 2022/143).

## 3. Results

The sociodemographic characteristics of the participants are presented in Table 1. A total of 295 nurses participated in the study. By gender, 72.2% of the nurses were female; 78.0% were graduates; 59.7% were single; 78.3% of the nurses had chosen their profession voluntarily; 87.5% had chosen to work in the emergency department and 78.6% of the nurses worked shifts.

**TABLE 1:** Distribution of nurses according to certain sociodemographic characteristics.

Category	Groups	*n* (%)
Gender	Female	213 (72.2)
	Male	82 (27.8)

Educational status	Health Vocational High School	23 (7.8)
	Associate’s degree	29 (9.8)
	Bachelor’s degree	230 (78.0)
	Postgraduate degree	13 (4.4)

Marital status	Married	119 (40.3)
	Single	176 (59.7)

Having children	Yes	89 (30.2)
	No	206 (69.8)

Desire to continue working as a nurse	Yes	231 (78.3)
	No	64 (21.7)

Type of hospital	Training and Research Hospital‐4	47 (15.9)
	Training and Research Hospital‐1	23 (7.8)
	Public Hospital‐1	52 (17.6)
	Training and Research Hospital‐2	29 (9.8)
	Training and Research Hospital‐3	23 (7.8)
	Public Hospital‐2	47 (15.9)
	University Hospital	74 (25.1)

Willingness to work in the unit	Yes	258 (87.5)
	No	37 (12.5)

Working pattern	Day shift only	57 (19.3)
	Night shift only	6 (2.0)
	Shift work	232 (78.6)

Table 2 presents the means of the scales, findings of normal distribution and reliability findings. The skewness and kurtosis values of the scales being within the ±1.5 range indicate that the data meet the assumption of normal distribution and that the use of parametric analyses is appropriate [33].

**TABLE 2:** Descriptive findings for intent to leave, impulsivity, anger and anger styles, cognitive control and flexibility scales.

	Mean	SD.	Min.	Max.	Kurtosis	Skewness	*α*
IMEAN	2.72	0.42	1.67	4.00	1.20	0.75	0.713
AMEAN	2.17	0.28	1.50	3.06	0.08	0.45	0.728
CMEAN	3.99	1.23	1.22	6.56	1.27	−0.191	0.908
ILMEAN	1.76	1.02	1.00	5.00	−0.929	1.20	0.884

*Note:* IMEAN: Impulsivity Scale Mean; AMEAN: Anger Scale Mean; CMEAN: Cognitive Control and Flexibility Scale; ILMEAN Intention to Leave.

Nurses were found to have high levels of cognitive control and flexibility, but low levels of intention to leave their jobs (Table 2). The reliability coefficients (*α* = 0.71–0.90) are acceptable and high, indicating that the scales provide reliable measurements in the sample.

The results of the multiple linear regression analysis, conducted to determine the predictors of intention to leave the job, are presented in Table 3. The model significantly explains 5.3% of the total variance in the intention to leave (*F* (3.196) = 6.499, *p* < 0.001, adjusted R2 = = 0.053). The Durbin–Watson value of 1.702 indicates the absence of autocorrelation, while VIF values between 1.01 and 1.06 suggest that there is no issue with multicollinearity among the independent variables. An examination of the regression coefficients reveals that impulsivity (*β* = 0.138, *t* = 2.366, *p* = 0.019, 95% CI: [0.048_0.524]), anger expression style (*β* = 0.131, *t* = 2.3302, *p* = 0.022, 95% CI: [0.068_0.866]) and cognitive control/flexibility (*β* = 0.121, *t* = 2.073, *p* = 0.039, 95% CI: [−0.196_−0.005]) are significant predictors of the intention to leave. These results suggest that cognitive control and flexibility are protective factors in terms of emotional regulation and stress management.

**TABLE 3:** The effect of impulsivity, anger expression, cognitive control and flexibility on intention to leave the job.

Independent variable	Unstandardized coefficients		Standardized coefficients	t	*p*	95% Confidence interval		Collinearity statistic	
	B	SE	ß			Lower	Upper	Tolerance	VIF
Constant	0.337	0.664							
IMEAN	0.286	0.121	0.138	2.366	0.019	0.048	0.524	0.952	1.051
AMEAN	0.467	0.203	0.131	2.302	0.022	0.068	0.866	0.987	1.013
CMEAN	0.100	0.048	−0.121	2.073	0.039	−0.196	−0.005	0.940	1.064

*Note:* Dependent variable = Intention to Leave. Adjusted *R* ^2^ = 0.053; *F* = 6.499; *p* ∼ 0.001; Durbin–Watson = 1.702; IMEAN: Impulsivity Scale Mean; AMEAN: Anger Scale Mean; CMEAN: Cognitive Control and Flexibility Scale Mean.

Correlation analyses support the regression findings (Table 4). A positive relationship was found between impulsivity and intention to leave the job (*r* = 0.167). A positive relationship was found between anger expression level and intention to leave the job (*r* = 0.149). A negative relationship was found between cognitive control and flexibility and intention to leave the job (*r* = −0.166).

**TABLE 4:** Correlation analyses of impulsivity, anger expression, cognitive control and flexibility in relation to intention to leave the job.

	IMEAN	AMEAN	CMEAN
AMEAN	0.025		
CMEAN	−0.219^∗∗^	−0.114	
ILMEAN	0.167 ^∗∗^	0.149^∗^	−0.166^∗∗^

^∗^ *p* ≤ 0.005.

^∗∗^ *p* ≤ 0.001.

As shown in Table 5, a positive relationship has been found between age, length of service in the profession and intention to leave the job. There is a positive relationship between nurses’ ages, length of service in the profession, their voluntary status within the unit and their impulsivity. A negative relationship was observed between having children and voluntarily choosing the nursing profession and the intention to leave the job.

**TABLE 5:** Relationships between scales and sociodemographic characteristics.

	Age	Choosing the nursing profession voluntarily	Length of service in the profession	Working voluntarily in the unit	Having children
IAN	0.196^∗∗^	−0.234^∗∗^	0.213^∗∗^	0.105	−0.155^∗∗^
DMEAN	0.125^∗^	0.061	0.127^∗^	0.125^∗^	−0.054
ÖMEAN	−0.034	0.086	−0.040	0.006	0.066
BMEAN	0.006	−0.182^∗∗^	−0.012	−0.072	0.081

^∗^ *p* ≤ 0.005.

^∗∗^ *p* ≤ 0.001.

## 4. Discussion

This study examined the relationships between impulsivity, cognitive control and flexibility on emergency department nurses’ intentions to leave their jobs. Although the variance explained in the findings was relatively small (*R* ^2^ = 5.3%) suggesting that additional organisational and environmental factors likely contribute to turnover intention, the results suggest that individual psychological characteristics are associated with nurses’ decisions to continue working or resign, particularly in high‐stress clinical settings such as emergency departments. It is expected that nurses working in challenging and complex work environments such as emergency departments would have high levels of cognitive assessment and coping. This is because cognitive flexibility is defined as an important psychological resource that facilitates individuals’ successful adaptation to environmental changes and supports coping with stressful situations [8, 34, 35].

One of the study’s key findings is the positive association between impulsivity and intention to leave. Nurses with high levels of impulsivity are more likely to report stronger intentions to leave their jobs. Impulsivity is generally associated with reduced behavioural regulation and a tendency to react quickly to stressful or unsatisfactory situations. It has also been reported that impulsivity is a heterogeneous personality trait encompassing various sub‐features such as risk‐taking, novelty seeking, sensation seeking and behavioural control weakness [36]. In the context of emergency departments, where rapid decision‐making and intense emotional demands are common, individuals with high impulsivity may experience greater difficulty coping with workplace stressors. Therefore, emergency department nurses may be more likely to report higher intention to leave when faced with persistent work‐related difficulties. Indeed, Kruczek and colleagues reported that cognitive flexibility and coping skills in nurses are positively associated with psychological adjustment [7]. The study revealed that anger expression was positively associated with the intention to leave work. Emergency departments are extremely challenging environments, characterised by time pressure, heavy workloads and frequent interaction with demanding patients and their relatives. Under these conditions, anger can emerge as a natural emotional response. However, when anger is expressed frequently or ineffectively, it can negatively impact interpersonal relationships, teamwork and job satisfaction. It should be noted, however, that the observed associations were modest in magnitude, and caution is warranted in interpreting their practical significance. Indeed, factors influencing turnover intention among emergency department nurses include understaffing, high physical workload, patient characteristics, inadequate pay, limited career development and burnout [37–39]. Over time, these challenges can increase nurses’ psychological stress and contribute to the development of their intention to leave. Furthermore, Park and Song [40] reported that resilience and violence perpetrated by patients are important factors affecting the intention to leave among emergency department nurses.

These findings indicate that the intense stress and adverse working conditions encountered in emergency departments are associated with nurses’ psychological well‐being and intention to leave, although causal directionality cannot be established given the cross‐sectional design. A negative association was found between cognitive control and cognitive flexibility and intention to leave, while a positive association was found with impulsivity and anger expression. Nurses with higher levels of cognitive flexibility and control reported lower intentions to leave their jobs. Within the framework of the JD‐R Model, cognitive flexibility and cognitive control may be conceptualised as personal psychological resources that buffer the adverse effects of high job demands on turnover intention [21].

Cognitive flexibility refers to the ability to adapt thinking and behaviour in response to changing environmental demands, while cognitive control involves the capacity to regulate attention, emotions and behavioural responses. Therefore, stressful working conditions may lead to coping difficulties in individuals with low cognitive control and flexibility. It may be speculated that this situation could contribute to maladaptive anger expression and interpersonal difficulties within the healthcare team, although these pathways were not directly measured in the current study [41]. Higher levels of cognitive regulation may be associated with a greater capacity to reinterpret stressful events, generate alternative solutions and maintain emotional balance in challenging situations. Nurses’ cognitive capacities may function as potential buffering resources that reduce the likelihood of developing intentions to leave the profession. Correlational findings further support this interpretation. Impulsivity was found to be negatively associated with cognitive control and flexibility, and positively associated with intention to leave the profession. This suggests that individuals who tend to respond impulsively may have lower cognitive regulation capacities [36]. The study also revealed several significant associations between sociodemographic characteristics and the main study variables. Intent to leave work was positively associated with age and professional experience.

Although experienced nurses possess greater clinical expertise, prolonged exposure to challenging working conditions in emergency departments can lead to accumulated stress, fatigue and burnout. Previous studies have shown that emergency department nurses experience high levels of job stress and burnout and that this significantly increases their intention to leave their jobs [42, 43]. Nurses with children reported lower levels of intention to leave their jobs. This finding indicates that family responsibilities are among the important factors influencing the decision to remain in employment. Family obligations and social ties can reduce the likelihood of employees leaving their jobs [44, 45].

The association between voluntarily choosing the nursing profession and the intention to leave the job is another important finding. Nurses who voluntarily chose the profession are less likely to report an intention to leave the job. The negative association between voluntarily choosing the profession and cognitive control and flexibility is an unexpected finding. Two theoretically coherent mechanisms may help explain this unexpected finding. First, nurses who enter the profession with strong idealistic motivation may be particularly vulnerable to reality shock, a well‐documented phenomenon in the nursing literature describing the dissonance between anticipated and actual working conditions [46]. When confronted with the high workload, emotional volatility and exposure to aggression typical of emergency departments, this mismatch may erode adaptive cognitive processes, including cognitive flexibility and coping, particularly when the gap between expectations and reality is substantial. Second, the finding may reflect a form of cognitive‐emotional dissonance, whereby a strong vocational identity coexists with chronic exposure to stressors that undermine that identity. This tension may transiently narrow cognitive flexibility by shifting attentional resources toward frustration, unmet expectations or perceived loss of professional meaning. Indeed, one study reported that the responsibility subscale of the reality shock scale explained 10.5% of the variance in intention to leave [47]. Future longitudinal studies should examine whether cognitive flexibility declines over time among nurses with high initial intrinsic motivation, and whether organisational factors such as leadership, staffing and perceived support moderate this trajectory.

The findings of this study contribute to the growing literature emphasising the importance of psychological and cognitive factors in workforce sustainability in healthcare settings. Organisational factors such as workload, leadership and working conditions are widely accepted determinants of turnover intentions [42, 44]. However, our findings indicate that nurses’ individual cognitive and emotional regulation capacities are also associated with their intention to leave their jobs. Given the modest effect sizes observed (*R* ^2^ = 5.3%), these findings should be interpreted as complementary to, rather than replacements for, broader organisational strategies. These findings offer important implications for practice. Interventions aimed at strengthening nurses’ cognitive flexibility and stress management skills may help reduce their intention to leave and contribute to retaining the workforce in emergency departments. Training programmes focusing on resilience, cognitive restructuring and emotional regulation strategies could be important organisational tools for healthcare institutions aiming to improve staff well‐being and organisational stability. Well‐structured training programmes for nurses may also help them stay in their jobs. A recent systematic review showed that nurses supported by such training improved not only their technical skills but also their clinical decision‐making and their ability to organise care [48]. By strengthening nurses’ professional role and autonomy, these training programmes may complement the cognitive and emotional skills highlighted in this study and support nurses’ retention in the profession.

### 4.1. Limitations

This study has certain limitations that should be considered when interpreting the findings. First, the cross‐sectional design prevents the establishment of causal relationships between the variables examined. Longitudinal studies are needed to clarify the directionality and stability of the observed associations over time. Second, data were collected exclusively through self‐report measures, which introduces the risk of response biases such as social desirability and common method bias. These biases may have influenced participants’ responses, particularly regarding sensitive constructs such as anger expression and turnover intention. Third, purposive sampling based on voluntary participation may have introduced selection bias, as nurses who agreed to participate may differ systematically from those who did not. This possibility should be considered when evaluating the representativeness of the sample. Fourth, the relatively low explained variance of the regression model (*R* ^2^ = 5.3%) suggests that the psychological variables examined in this study account for only a modest proportion of the variance in turnover intention. Organisational, environmental and occupational factors such as workload, staffing levels, leadership quality and institutional support may substantially contribute to nurses’ intention to leave and warrant investigation in future studies. Finally, the study was conducted in emergency departments located in a single geographical area, which may limit the generalisability of the findings to other clinical settings, regions or healthcare systems.

Despite these limitations, this study has several notable strengths. It focuses specifically on emergency department nurses, a population that remains underrepresented in the nursing turnover literature. Furthermore, it simultaneously examines multiple psychological variables including cognitive flexibility, cognitive control, impulsivity and anger expression in relation to turnover intention, thereby offering a more comprehensive perspective than studies addressing these constructs in isolation. The findings provide practical insights relevant to nursing management and workforce retention strategies in high‐pressure clinical environments.

## 5. Conclusion

This study focused on psychological resources that have been addressed only to a limited extent in the literature, examining the effects of cognitive flexibility, cognitive control, impulsivity and anger expression styles on turnover intention among emergency department nurses. The findings reveal that cognitive control and cognitive flexibility are negatively associated with intention to leave, whereas impulsivity and anger expression levels are positively associated with turnover intention. These results reveal that nurses’ cognitive and emotional regulation capacities are important individual resources that shape their intention to leave in emergency department settings with high job demands.

Research findings indicate that cognitive control and cognitive flexibility were associated with greater capacity to regulate emotional responses and adapt to changing work conditions in stressful work environments. Conversely, higher levels of impulsivity and anger expression were associated with greater turnover intentions, particularly in emergency department settings characterised by heavy workloads and time pressure. This supports the notion that individual psychological resources are important factors that may buffer the negative effects of job demands.

When evaluated in terms of demographic and occupational variables, it was observed that having children and voluntarily choosing one’s profession were associated with a reduction in the intention to leave work. Conversely, the positive relationship between increasing age and length of service in the profession and the intention to leave work suggests that long‐term occupational burdens may increase employees’ thoughts of leaving work. Furthermore, the unexpected negative relationship between voluntarily choosing the profession and cognitive control and flexibility can be evaluated within the framework of the concept of “reality shock,” which is frequently discussed in the nursing profession. In general, this study demonstrates that emergency department nurses’ intention to leave their jobs is not solely dependent on organisational and working conditions; it is also closely associated with individual cognitive and emotional regulation skills. In this regard, planning training and support programmes in healthcare institutions to develop nurses’ cognitive flexibility, emotional regulation and stress management skills may strengthen employees’ tendency to remain in their jobs. Particularly in units with high work demands, such as emergency departments, supporting individual resources that enhance psychological resilience may contribute to both improving employee well‐being and ensuring the sustainability of healthcare services.

## Author Contributions

Nukhet Bayer and Ayşegül Turan contributed to the conceptualisation and design of the study. Nukhet Bayer undertook data collection, provided study resources and performed data curation. Ayşegül Turan conducted the formal statistical analysis and prepared the figures. Nukhet Bayer wrote the original draft; both authors contributed to the review and editing of the manuscript and provided supervision.

## Funding

This study did not receive any financial support.

## Disclosure

All authors approved the final version of the manuscript.

## Ethics Statement

Approval for the research was obtained from the Non‐Interventional Clinical Research Ethics Committee of Lokman Hekim University (Decision No: 2022/143 and Code No: 2022131). Written permission was obtained from the hospital administrations where the study was conducted. Nurses invited to participate in the research were provided with information about the research, and those who gave their consent to participate voluntarily were included in the study. The nurses’ personal details were not recorded on the data collection forms.

## Conflicts of Interest

The authors declare no conflicts of interest.

## Supporting Information

Additional supporting information can be found online in the Supporting Information section.

## Supporting information


**Supporting Information** STROBE Statement—Checklist of items that should be included in reports of cross‐sectional studies.

## Data Availability

The data that support the findings of this study are available upon request from the corresponding author. The data are not publicly available due to privacy or ethical restrictions.
